# Precision oncology for intrahepatic cholangiocarcinoma in clinical practice

**DOI:** 10.1038/s41416-022-01932-1

**Published:** 2022-08-19

**Authors:** Aurelie Tomczak, Christoph Springfeld, Michael T. Dill, De-Hua Chang, Daniel Kazdal, Ursula Wagner, Arianeb Mehrabi, Antje Brockschmidt, Tom Luedde, Patrick Naumann, Albrecht Stenzinger, Peter Schirmacher, Thomas Longerich

**Affiliations:** 1 Institute of Pathology, Heidelberg University Hospital, Heidelberg, Germany; 2 Liver Cancer Centre Heidelberg, Heidelberg, Germany; 3 Medical Oncology, National Centre for Tumor Diseases, Heidelberg, Germany; 4 Department of Gastroenterology, Infectious Diseases, Intoxication, Heidelberg University Hospital, Heidelberg, Germany; 5 Experimental Hepatology, Inflammation and Cancer Research Group, German Cancer Research Centre (DKFZ), Heidelberg, Germany; 6 Department of Diagnostic and Interventional Radiology, Heidelberg University Hospital, Heidelberg, Germany; 7 Department of General, Visceral & Transplantation Surgery, Heidelberg University Hospital, Heidelberg, Germany; 8 Clinical Cancer Registry, National Centre for Tumor Diseases, Heidelberg, Germany; 9 Clinic for Gastroenterology, Hepatology and Infectious Disease, University Hospital Düsseldorf, Düsseldorf, Germany; 10 Department of Radiation Oncology, Heidelberg University Hospital, Heidelberg, Germany

**Keywords:** Molecular medicine, Bile duct cancer

## Abstract

**Background:**

Advanced cholangiocarcinoma has a poor prognosis. Molecular targeted approaches have been proposed for patients after progression under first-line chemotherapy treatment. Here, molecular profiling of intrahepatic cholangiocarcinoma in combination with a comprehensive umbrella concept was applied in a real-world setting.

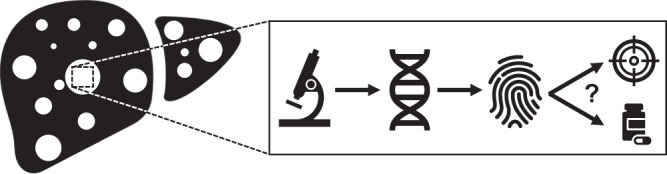

**Methods:**

In total, 101 patients received molecular profiling and matched treatment based on interdisciplinary tumour board decisions in a tertiary care setting. Parallel DNA and RNA sequencing of formalin-fixed paraffin-embedded tumour tissue was performed using large panels.

**Results:**

Genetic alterations were detected in 77% of patients and included gene fusions in 21 patients. The latter recurrently involved the *FGFR2* and the *NRG1* gene loci. The most commonly altered genes were *BAP1*, *ARID1A*, *FGFR2*, *IDH1*, *CDKN2A*, *CDKN2B*, *PIK3CA*, *TP53*, *ATM*, *IDH2*, *BRAF*, *SMARCA4* and *FGFR3*. Molecular targets were detected in 59% of patients. Of these, 32% received targeted therapy. The most relevant reason for not initiating therapy was the deterioration of performance status. Patients receiving a molecular-matched therapy showed a significantly higher survival probability compared to patients receiving conventional chemotherapy only (HR: 2.059, 95% CI: 0.9817–4.320, *P* < 0.01).

**Conclusions:**

Molecular profiling can be successfully translated into clinical treatment of intrahepatic cholangiocarcinoma patients and is associated with prolonged survival of patients receiving a molecular-matched treatment.

## Introduction

Anatomically, three types of biliary tract cancer are distinguished. These include intrahepatic cholangiocarcinoma (iCCA), extrahepatic CCA (eCCA), and gallbladder cancer [1, 2]. While the latter group of tumours develop via precursor lesions (e.g., biliary intraepithelial neoplasia), the cellular origin of iCCA appears to be diverse and may be associated with the histological subtype. In particular, the small-duct type of iCCA has been observed as a component of combined hepatocellular cholangiocarcinoma and experimental data from mice suggest that iCCA may develop from hepatocytes [3]. The block of hepatocellular differentiation due to epigenetic inactivation of Hepatocyte Nuclear Factor-4α expression in liver cells expressing mutant isocitrate dehydrogenase (IDH) 1 or 2 has been identified as one underlying mechanism [4]. In line with this, the molecular landscape of human iCCA includes a subgroup of tumours with oncogenic *IDH1* and *IDH2* mutations; genetic variants which are not observed in extrahepatic biliary tract tumours [5].

Most CCA patients are diagnosed with advanced disease. Based on the findings of the ABC-02 and BT22 studies, combined cisplatin and gemcitabine (CISGEM) treatment improved the median overall survival from 8.0 to 11.6 months compared to gemcitabine monotherapy and remained the standard first-line systemic therapy for more than a decade [6]. Recently, pre-planned interim analysis of the TOPAZ-1 trial showed improved overall (12.8 vs. 11.5 months, *P* = 0.021) and progression-free survival (7.2 vs. 5.7 months, *P* = 0.001) in patients with advanced biliary tract cancer treated with the PD-L1 inhibitor durvalumab plus CISGEM compared to placebo plus CISGEM [7]. In addition, the ABC-06 study established 5-fluorouracil/oxaliplatin (FOLFOX) as second-line chemotherapy [8]. Despite these advances, the prognosis of iCCA patients remains poor. Consequently, molecularly targeted approaches have been proposed for patients with good performance status after at least one previous conventional systemic chemotherapy regimen. Meta-analyses showed that such a biomarker-based selection strategy was associated with significantly improved response and survival rates in Phase 1 and in Phase 2 clinical trials in diverse cancer entities [9, 10].

A recent multicentre, randomised, double-blind, placebo-controlled, Phase 3 study (ClarIDHy) demonstrated that progression-free and overall survival were significantly improved with ivosidenib, a small-molecule targeted inhibitor of mutated IDH1, in patients with previously treated IDH1-mutant CCA compared to placebo and subsequently received FDA approval (reference ID: 442715) [11, 12]. Another recurrent molecular feature of iCCA is the presence of gene fusions [5]. Here, the FIGHT-202 study showed that treatment with pemigatinib, a selective, oral inhibitor of fibroblast growth factor receptor (FGFR) 1, 2 and 3, resulted in an objective response in previously treated CCA patients with detectable *FGFR2* gene rearrangements; [13] a finding recently leading to authorisation of pemigatinib monotherapy for the treatment of adults with locally advanced or metastatic cholangiocarcinoma with the presence of a *FGFR2* gene rearrangement that has progressed after at least one prior line of systemic therapy (EMEA/H/C/005266). In addition, infigratinib has been licensed (EU/3/20/2329) and futibatinib among others showed objective response rates in about 25% of patients with fusion-positive iCCA in Phase 2 studies as well [14, 15]. Thus, there is a continuously evolving landscape of clinically relevant FGFR inhibitors.

Within the MOSCATO-01 trial, a targeted treatment based on molecular tumour profiling resulted in improved survival of patients suffering from biliary tract cancers [16]. However, it remained elusive whether such an approach may also be feasible in a real-world setting. In 2018, the Liver Cancer Centre Heidelberg (LCCH) has initiated an umbrella concept for patients with advanced liver cancer: All patients are discussed by the interdisciplinary tumour board and a large panel-based molecular profiling of liver cancer samples is performed for patients, who are considered eligible for systemic treatment. This approach is intended to fulfil three major aims: (i) facilitating molecular targeted therapy in daily practice, (ii) enrichment of clinical studies with patients, whose tumours carry a genetic alteration likely to respond to the drug under investigation and (iii) a personalised treatment decision in the setting of more than one treatment option and/or available drugs approved in other entities. Since November 2021 LCCH patients can also be referred to the Centre for Personalised Medicine Heidelberg, thereby providing a framework for inclusion into molecularly stratified studies and initiation of off-label therapies based on decisions of a molecular tumour board.

## Materials and methods

### Human tissue samples

Patients with primary liver cancer referred to our hospital were enrolled in the LCCH registry study, which was approved by the local ethics committee of the Heidelberg University Hospital (S-693/2019). Informed consent was obtained from all prospectively enrolled patients. All patients receiving molecular testing were discussed by the interdisciplinary LCCH tumour board and considered eligible for systemic tumour therapy. The main inclusion criteria were an ECOG performance status of 0 or 1, an advanced (unresectable, locally advanced or metastatic) primary liver cancer treated by at least one line of systemic chemotherapy, and available formalin-fixed, paraffin-embedded (FFPE) tumour tissue (either from prior tumour resection or diagnostic biopsy). The patients reported here received molecular profiling between January 2018 and March 2021. The data cut-off date for the final analysis was December 14, 2021. The cohort includes 96 intrahepatic cholangiocarcinoma (iCCA) cases, four combined hepatocellular-cholangiocarcinomas and one undifferentiated primary liver carcinoma. Histological samples were all reviewed by two expert hepatopathologists (TL and PS) and all liver carcinomas were classified according to the current WHO classification [1, 17]. The main patient´s characteristics are shown in Supplementary Table S1.

### DNA extraction and quantification

Briefly, tumour tissue was microdissected to achieve sufficient histological tumour content. All samples had a tumour content >10%. For DNA extraction, six consecutive 10-μm-thick FFPE sections of each sample were pooled, deparaffinized and digested with proteinase K overnight. The extraction was performed with an automated Maxwell 16 Research extraction system (Promega, Madison, WI, USA) using the Maxwell^®^ 16 FFPE Plus LEV RNA Purification Kit, following the manufacturer’s instructions. The sample was split for DNA and RNA extraction. To obtain DNA-free RNA the TURBO DNA-free™ Kit (Thermo Fisher Scientific, Waltham, MA, USA) was used to digest the DNA. The concentrations of DNA and RNA were measured fluorometrically (QuBit 2.0 DNA respectively RNA high sensitivity kit; Thermo Fisher Scientific). To quantify the amount of amplifiable nucleic acids a qPCR assay (RNAseP assay, Thermo Fisher Scientific) was used.

### Library preparation and massive parallel sequencing

#### Oncomine comprehensive assay v3 (OCAv3)

To prepare the OCAv3 (Thermo Fisher Scientific) sequencing library the multiplex polymerase chain reaction (PCR)-based Ion Torrent AmpliSeqTM technology was used as described previously [18]. In brief, amplicon library preparation was performed using the Ion AmpliSeq Library Kit v2.0 and ~10 ng of input DNA. Briefly, the DNA was mixed with the two primer pools (containing all primers for generating the 3781 amplicons) and the AmpliSeq HiFi Master Mix before they were transferred to a PCR cycler (BioRad, Munich, Germany). For detection of gene fusions, RNA was reverse transcribed using the SuperScript™ VILO™ cDNA Synthesis Kit according to the manufacturer’s instructions (Thermo Fisher Scientific). The amplicon libraries were prepared from 20 ng RNA, which were mixed with two primer pools generating 1732 amplicons and the AmpliSeq HiFi Master Mix before transferring them to a PCR cycler (BioRad). Subsequently, DNA, respectively, RNA pools were combined and primer end sequences were partially digested using FuPa reagent, followed by the ligation of barcoded sequencing adapters (Thermo Fisher Scientific). The final libraries were purified using AMPure XP magnetic beads (Beckman Coulter, Krefeld, Germany) and quantified by qPCR (Ion Library Quantitation Kit, Thermo Fisher Scientific) using a StepOne qPCR machine (Thermo Fisher Scientific).

The individual libraries were diluted to a final concentration of 100 pM and six samples (every six libraries for mutation and gene fusion detection) were pooled and processed to library amplification using Ion Spheres and the Ion 530 Chef Kit for library enrichment on the Ion Chef System (Thermo Fisher Scientific). The libraries were then processed for sequencing using the Ion S5 Sequencing chemistry and the barcoded libraries were loaded onto a 530-chip. Pooling six samples on a 530-chip resulted in a mean coverage of 1000-fold per amplicon.

#### TruSight Oncology 500 (TSO500)

In the initial step of the capture-based TSO500 (Illumina, San Diego, CA, USA) library preparation, the DNA integrity status of a sample was determined by Genomic DNA ScreenTape Analysis using a 4150 TapeStation System (both Agilent, Santa Clara, CA, USA). 80 ng of total DNA of each sample were sheared according to their degradation level for 50–78 s using a focused ultrasonicator ME220 (Covaris, Woburn, MA, USA) to generate DNA fragments ranging between 90 and 250 bp. Following two-target capture and purification, the enriched libraries were amplified (15 PCR cycles) and subsequently quality controlled using the KAPA SYBR Library Quantification Kit on a StepOnePlus quantitative PCR system (both Thermo Fisher Scientific). Up to eight libraries were sequenced simultaneously using a NextSeq 500 system (Illumina) with a high-output cartridge and v2 chemistry. All assays were performed according to the manufacturer's protocols [19].

#### TruSight Tumor 170 (TST170) RNA assay

For enriched cDNA library preparation using the TST170 RNA assay, hybrid capture-based library preparation was applied as described by the manufacturer (Illumina). In brief, 50–100 ng RNA was applied to cDNA conversion (first-strand and second-strand synthesis). The library preparation comprised end repair, adenylation, adaptor ligation, and index PCR. For enrichment of the libraries, biotinylated probes were hybridised and captured twice using streptavidin-coated beads. After amplification of the enriched libraries, quantification and pooling of libraries were done, and libraries were sequenced on a NextSeq 500 system (Illumina).

#### Anchored multiplex PCR (AMP)-based NGS translocation detection

Anchored Multiplex PCR (AMP)-based translocation detection represents an NGS technology, which uses unidirectional gene-specific primers to facilitate open-ended amplification and thus the detection of novel rearrangements in RNA derived from FFPE samples in combination with molecular barcodes to enable unique molecule counting and error correction. For enriched cDNA library preparation, the multiplex PCR-based Ion Torrent AmpliSeqTM technology (Thermo Fisher Scientific) with the Archer® FusionPlex^®^ Solid Tumor assay (ArcherDX, Boulder, CO, USA) was used according to the manufacturer’s instructions. In short, 100-200 ng RNA was reverse transcribed for first-strand synthesis and subjected to real-time PCR (Archer PreSeq RNA QC assay). Only samples with a QC score ≤30 were used for analysis. Hereafter, a 2nd strand synthesis was performed, followed by product end repair, phosphorylation and adenylation followed by ligation of the Ion Torrent specific MBCv2 adapters resulting in half functional molecular barcoded double-strand cDNA. The first PCR was performed with anchored gene-specific primers covering 53 target genes and a universal primer located at the end of the MBCv2 adapter. The second PCR with nested gene-specific primers carrying the index for sample multiplexing and the universal primers produces the fully functional library, which was quantified using a StepOne qPCR device (Ion Library Quantitation Kit, Thermo Fisher Scientific). Only libraries with ≥10.000 pM were used for further analysis. The individual libraries were diluted to a final concentration of 50 pM. Samples were pooled and processed to library amplification using Ion Spheres and the Ion 530 or 540 Chef kits Chef kits for library enrichment on the Ion Chef System (Thermo Fisher Scientific). The libraries were then processed for sequencing using the Ion S5 Sequencing chemistry. The barcoded libraries were loaded onto 530 (max. 5 libraries) or 540 (max. 14 libraries) chips to achieve a minimum amount of 3 million reads per sample. Data were analysed using the Archer analysis software (Version 5.1) for the presence of gene fusions. The sequence quality of each sample was assessed by the following criteria: >10% or at least 150.000 unique fragments, more than 50 average unique RNA start sites per GSP2 control and a target deduplication ratio of less than 40. If all criteria were met but no specific fusion was detected, the sample was considered negative for fusions detectable with the assay-specific primers.

### Variant calling and annotation

Data analysis of the OCAv3 panel was performed using the Ion Torrent Suite Software (version 5.8.0). After base calling, the reads were aligned against the human genome (Genome Reference Consortium Human Build 37) using the TMAP algorithm within the Torrent Suite. Variant calling was performed with the variant caller plugin (version 5.8.7-1) within the Torrent Suite Software and the IonReporter package using a corresponding bed-file containing the coordinates of the amplified regions.

Concerning the TSO500 panel, a procession of raw sequencing data and variant calling was carried out using the TruSight Oncology 500 Local App (Illumina, pipeline version 1.3.0.39). To reduce artefacts, the presence of a variation called in one sample of a respective patient was checked in all associated samplesVariant annotation was performed as described previously [18, 20]. Only variants with an allele frequency above 2% and minimum coverage of greater than 100-fold were considered [21]. Annotations included information about nucleotide and amino acid changes of RefSeq annotated genes, COSMIC and dbSNP entries, as well as detection of possible splice site mutations. For data interpretation and verification, the aligned reads were visualised using the Integrative Genomics Viewer browser [22].

### Survival analyses

Overall survival was calculated from the date of diagnosis to the event of death by any cause. Survival time was censored for patients, who did not experience the investigated event. The association between survival and application of targeted tumour therapy was represented using a Kaplan–Meier plot and quantified by Gehan–Breslow–Wilcoxon test, hazard ratio (HR) and 95% confidence interval (CI) based on a log-rank test. The non-parametric Wilcoxon rank-sum test was used to analyse whether the presence of a certain genetic alteration was related to the treatment response (time-to-progression) towards conventional chemotherapy. Statistical analyses were implemented using GraphPad prism 9 (GraphPad Software, San Diego, CA, USA). *P* < 0.05 was considered statistically significant.

## Results

### Molecular profiling of liver cancer patients

A total of 101 consecutive patients were evaluated. The cohort included 96 iCCA, four HCC-CCA, and one undifferentiated primary liver cancer. Fifty-four percent of the patients were male and the median age at disease diagnosis was 60 years (Supplementary Table S1). The molecular analyses evolved over time and were in some cases also dependent on the quality of the histopathological samples.

OCAv3 allows for the interrogation of 161 genes (mainly hotspot regions or several exons) at the DNA level, while TSO500 covers 523 genes (full exonic coverage) [19]. Clinically relevant advantages of the latter panel are the additional possibilities to estimate tumour mutational burden, to predict microsatellite instability, and to detect a higher number of copy number alterations. Noteworthy, less DNA input is required for OCAv3, which may thus be an advantage for the evaluation of biopsy samples with minor tumour content. DNA sequencing data were available for 73% (*n* = 74/101) of patients and were generated using the OCAv3 panel in 60% (*n* = 61/101) and the TSO500 panel in 15% (*n* = 15/101) of patients.

Regarding gene fusions, which were analysed in all patients, there are important differences between the assays used [23]. OCAv3 is an amplicon-based approach and can detect only those fusions that were considered during panel design. In contrast, AMP-based translocation detection relies on single-primer extension and are capable of detecting gene fusions with yet unknown fusion partners. TST170 constitutes a hybrid capture-based assays, which captures targeted RNA molecules regardless of the neighbouring nucleotide sequence and is thus also able to detect unknown partner genes. These technical considerations have particular relevance for the evaluation of FGFR2 fusions, for which more than 150 partner genes have been reported in the literature until now [24]. In this study, AMP, OCAv3, and TST170 panels were applied in 78% (*n* = 79/101), 21% (*n* = 21/101), and 15% (*n* = 15/101) of patients, respectively (Supplementary Fig. S1).

While DNA sequencing was successful for all patients analysed, RNA sequencing for translocation detection failed in 6% of patients (*n* = 6/101) due to insufficient RNA preservation.

**Fig. 1:**
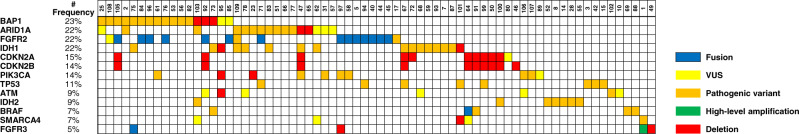
Most prevalent genomic alterations detected among patients with intrahepatic cholangiocarcinoma (*n* = 101). The given frequencies were calculated based on the number of patients with both available DNA and RNA sequencing data (*n* = 74). The type of alteration is colour-coded.

Genetic alterations were detected in 77% of patients (*n* = 78/101). In 21 patients, without any detected genetic alteration only RNA sequencing was performed. In fact, no genetic alteration was only found in two patients with both available DNA and RNA sequencing data. Gene fusions were observed in 21 patients (Supplementary Fig. S2) and recurrently involved the gene loci of *FGFR2* at chromosome 10q26.13 (*n* = 14) and *NRG1* at chromosome 8p12 (*n* = 2), respectively. One hundred fifty-six missense or nonsense variants were identified, 98 of which were considered likely pathogenic or pathogenic. The remaining events fulfilled the criteria of variants of unknown significance according to the ACMG/AMP joined consensus recommendations [25]. In addition, 66 whole gene deletions and 4 high-level amplifications were found (Supplementary Fig. S3 and Supplementary Table S2). The most commonly altered genes (Fig. 1) in tumour tissues were *BAP1* (23%), *ARID1A* (22%), *FGFR2* (22%), *IDH1* (22%), *CDKN2A* (15%), *CDKN2B* (14%), *PIK3CA* (14%), *TP53* (11%), *ATM* (9%), *IDH2* (9%), *BRAF* (7%), *SMARCA4* (7%) and *FGFR3* (5%). Registration office data on overall survival were available from 61 patients with available DNA and RNA sequencing data. In this sub-cohort, we did not observe a statistical association between survival and mutations in any of the most commonly altered genes mentioned above (*P* > 0.05).

### Targeted therapy in a real-world scenario

**Fig. 2:**
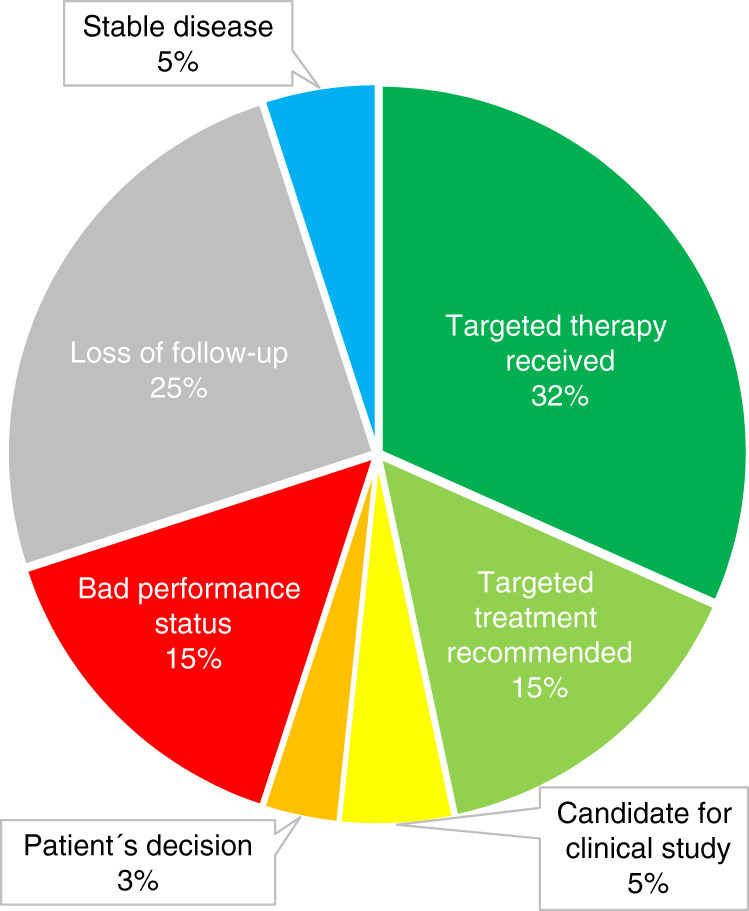
Efficacy of translating molecular profiling into patient’s treatment. The pie chart details the frequencies and reasons for receiving or not receiving a molecular-matched therapy.

Nineteen of the 82 genes (23%), in which genetic alterations were detected (Supplementary Table S2), were considered targetable (Supplementary Table S3). Overall, the sequencing analyses revealed molecular targets in 59% (*n* = 60/101) of patients. From these, 32% (n = 19/60) received targeted therapy (Fig. 2 and Table 1). The following drugs were applied: pemigatinib (*n* = 3) and derazantinib (*n* = 2) for *FGFR2* fusions (*n* = 5), olaparib for deleterious *BAP1* and *BRCA1* mutation, respectively (*n* = 2), dabrafenib plus trametinib for *BRAF* mutation (*n* = 2), enasidenib for *IDH2* mutation (*n* = 2), infigratinib for *FGFR3* fusion (*n* = 2), afatinib for *NRG1* fusion (*n* = 2), trastuzumab for low-level *ERBB2* amplification with HER2 score 2 + (*n* = 1), lenvatinib for *FGFR3* amplification (*n* = 1), crizotinib for *MET* rearrangement (*n* = 1), everolimus for *PIK3CA* mutation (*n* = 1), and pembrolizumab in one patient with known Lynch´s syndrome due to a *MSH2* germline mutation. In this last patient a complete and lasting remission was observed. A partial response was observed in five patients, leading to an objective response rate of 30% (*n* = 6/20 targeted treatments). The stable disease could be achieved in nine patients and three patients showed progressive disease at the first staging. In two patients, the best response could not be evaluated. Thus, the disease control rate was 75% (*n* = 15/20; Supplementary Table S4). In addition, targeted therapy was recommended in nine patients during admission for second opinion (15%). Five percent (*n* = 3/60) of patients were considered eligible candidates for a clinical study. Two patients (3%) decided against any further systemic tumour therapy.

**Table 1:** Targeted therapies applied to iCCA patients.

Pat. no.	Entity	Treatment lines (*n* = )	Molecular lesion	Treated with	Treatment stopped due to	Time-to-progression (d)
1	HCC-CCA	4	GOLGB1::MET fusion FGFR3 amplification	Crizotinib	Progressive disease	303
1	HCC-CCA	4	GOLGB1::MET fusion FGFR3 amplification	Lenvatinib	Progressive disease	89
5	iCCA	5	FGFR2::NOL4 fusion	Derazantinib	Progressive disease	102
8	iCCA	5	IDH2 (p.Arg172Gly)	Enasidenib	Progressive disease	113
10	iCCA	6	BRCA1 (p.Thr1561fs*40, p.Trp1815*)	Olabparib	Progressive disease	485
28	iCCA	4	IDH2 (p.Arg172Gly)	Enasidenib	Hyperbilirubinemia	NE
29	iCCA	3	MSH2 (p.Leu800Pro)	Pembrolizumab		>783
40	iCCA	2	FGFR2::TACC2 fusion	Derazantinib	Progressive disease	154
45	iCCA	7	FGFR2::BICC1 fusion	Infigratinib	Progressive disease	161
46	iCCA	4	ERBB2 low-level amplification	Trastuzumab	Progressive disease	195
58	iCCA	6	PIK3CA (p.Glu542Lys) FGFR2::CEP fusion (detected during sequencing of re-biopsy)	Everolimus	Progressive disease	304
63	iCCA	6	VTCN1::NRG1 fusion	Afatinib	Progressive disease	162
69	iCCA	4	BRAF (p.Val600Glu)	Dabrafenib/Trametinib		>286
74	iCCA	6	NRG1 (NOTCH2::NRG1)	Afatinib		>205
75	iCCA	5	FGFR3::TACC3 fusion BAP1 (p.spl?) IDH1 (p.Arg132Gly)	Infigratinib	Progressive disease	56
82	iCCA	3	BAP1 (p.spl?)	Olabparib		>282
84	iCCA	3	FGFR2::KIAA1217 fusion BAP1 (p.Lys425fs*5)	Pemigatinib	Progressive disease	216
88	iCCA	3	BRAF (p.Gly469Ala)	Dabrafenib/Trametinib		>92
94	iCCA	3	FGFR2::DBP fusion	Pemigatinib	Progressive disease	159
96	iCCA	7	FGFR2::BICC1 fusion BAP1 (p.Lys580fs*61, loss)	Pemigatinib		>163

*CR* complete response, *HCC-CCA* combined hepatocellular cholangiocarcinoma, *iCCA* intrahepatic cholangiocarcinoma, *NE* not evaluable.

Results of both DNA and RNA sequencing were available within a median of 9 working days (range 3–31) after request of molecular analysis; in particular 9 days for AMP (range 4–23), 7 days for OCAv3 (range 3–24), and 10 days for TSO500/TST170 (range 8–31). We did not detect a statistical difference in turn-around time between patients that could be implemented an NGS-informed therapy or not (median 10 vs. 9 days, *P* > 0.05).

The most relevant reason for not initiating a targeted therapy was a deteriorated performance status related to rapidly progressing tumour disease (15%, *n* = 9/60). Furthermore, 15 of 60 patients were lost to follow-up. The main reason for lost follow-up (87% of cases) was consultation for a second opinion and treatment at an external institution.

**Fig. 3:**
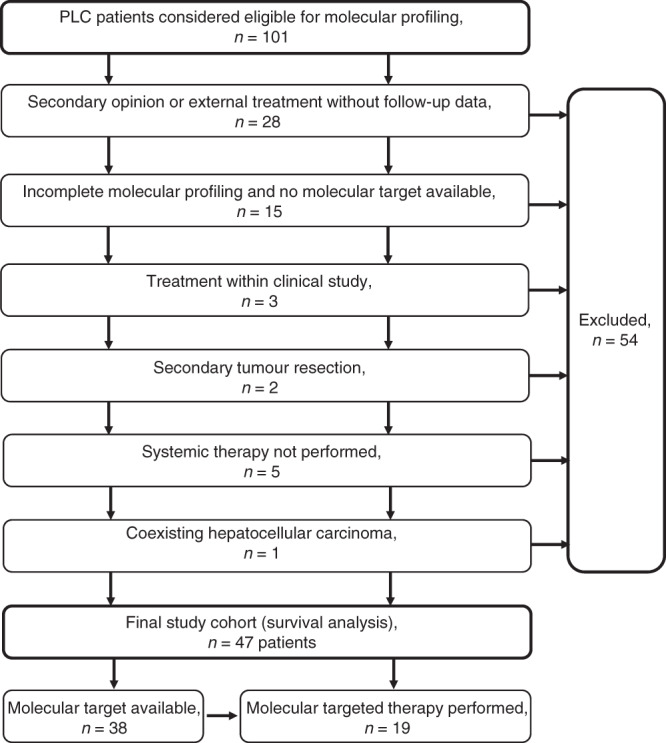
Flowchart of the patients sequenced within the LCCH umbrella. Finally, a molecular target was available in 38 out of 47 patients, from which 19 received a molecular-matched drug treatment.

**Fig. 4:**
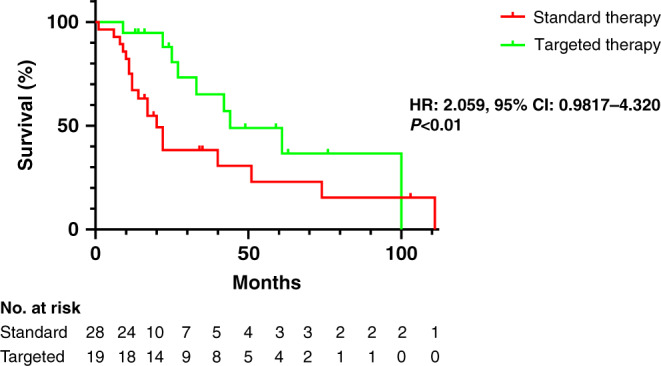
Impact of targeted treatment on overall survival. Kaplan–Meier curve of overall survival in patient’s receiving a molecular targeting drug compared to patients receiving standard treatment. The patients remaining at risk are detailed below the diagram. HR hazard ratio, CI confidence interval.

Forty-seven of the 101 patients could be evaluated regarding overall survival (Supplementary Table S4). The characteristics of this sub-cohort are shown in Table 2. Reasons for exclusion were external treatment without detailed clinical follow-up (*n* = 28), no molecular target in the setting of incomplete molecular profiling data (*n* = 15), the inclusion of patients into a clinical study (*n* = 3), secondary tumour resection following conventional chemotherapy (*n* = 2), systemic treatment not performed (*n* = 5), and coexisting hepatocellular carcinoma (*n* = 1; Fig. 3). Interestingly, patients in whom a targeted therapy was performed showed a significantly higher survival probability compared to patients receiving standard systemic therapy only (HR: 2.059, 95% CI: 0.9817–4.320, *P* < 0.01); Fig. 4). Of note, the number of treatment lines was significantly higher in patients receiving a molecularly targeted treatment compared to conventionally treated patients (median 4 vs. 2; *P* < 0.0001), while the number of detected druggable alterations was not different between both subgroups (*P* = 0.48).

**Table 2:** Patients´ characteristics of the survival cohort.

	Molecular targeted treatment (*n* = 19)	Standard treatment (*n* = 28)	Total (*n* = 47)
Median age (range)	51 (23–73)	62 (32–82)	58 (23–82)
Sex			
Male	8	15	23
Female	11	13	24
Tumour type			
iCCA	18	25	43
HCC-CCA	1	3	4
Systemic treatment lines (median, range)	4 (2–7)	2 (1–7)	3 (1–7)
Overall survival (months) (median, range)	27 (7–100)	17 (1–111)	20 (1–111)
Number of patients with ≥1 detected genetic alteration	19	24	42
Median number of druggable alterations per patient (range)			
iCCA	1 (1–3)	1 (0–6)	1 (0–6)
HCC-CCA	3	2 (1–2)	2 (1–3)

CISGEM treatment represents the current first-line treatment for biliary tract cancer for the last decade [6] and was used as first line in 28 patients. Six patients received gemcitabine-oxaliplatin (GEMOX) and two patients were initially treated with dose-adapted gemcitabine–carboplatin (GEMCARBO) due to renal insufficiency. Another four patients received fluorouracil–leucovorin–irinotecan–oxaliplatin (FOLFIRINOX) as the first systemic treatment as they were younger and downstaging may have allowed for a secondary surgical approach. FOLFOX was the primary regimen applied in two patients due to the presence of hyperbilirubinemia. CISGEM was supplemented by either durvalumab (*n* = 1) or durvalumab and tremelimumab (*n* = 1) within a study (NCT03473574). Two young patients received CISGEM with paclitaxel (*n* = 2) [26], one patient CISGEM with panitumumab as in the PiCCA study (NCT01320254).

Finally, we wanted to address whether particular mutated genes may be related to the treatment response towards conventional chemotherapy regimen. However, we did not observe a difference between response to CISGEM, FOLFIRINOX, FOLFOX or FOLFIRI polychemotherapy, respectively, and the presence or absence of mutations in the *ARID1A*, *ATM*, *BAP1*, *BRAF*, *CDKN2A*, *CDKN2B*, *FGFR2*, *FGFR3*, *IDH1*, *IDH2*, *PIK3CA*, *SMARCA4* and *TP53* genes (*P* > 0.05).

## Discussion

ICCA is frequently diagnosed at advanced disease stages; thus, palliative chemotherapy is the treatment of choice for many patients. The standard protocol CISGEM was applied as first-line treatment in most of our patients [6]. In individual cases, we decided to initiate a FOLFIRINOX polychemotherapy aiming at downstaging to achieve secondary resection as applied for pancreatic ductal adenocarcinoma [27]. Noteworthy, this treatment approach is no longer pursued, as the recently published PRODIGE 38 AMEBICA study showed that CISGEM is superior to FOLFIRINOX in iCCA patients [28]. Our analysis did not reveal specific genetic alterations that may be used to predict the treatment response of iCCA patients towards CISGEM, FOLFIRINOX, FOLFOX, and FOLFIRI chemotherapy regimen. However, we cannot rule out that such an association may exist, but at least in our cohort it was not observed.

In the setting of disease progression, molecularly targeted approaches have been proposed as an alternative treatment option for patients with good performance status. Within a clinical trial setting, a targeted treatment matching the molecular tumour profiling was already shown to improve the survival of patients suffering from biliary tract cancers [16]. However, it remained elusive whether such an approach may also be feasible in clinical practice. The current analysis of our ongoing LCCH umbrella concept showed for the first time an improvement of overall survival for iCCA patients receiving a molecularly matched, non-first-line treatment in a real-world scenario. Of note, the patients in the precision oncology group received significantly more lines of treatment compared to those receiving non-matched treatments only (median 4 vs. 2, *P* < 0.001). Nevertheless, patients in whom a targeted therapy could be initiated had a significantly better survival probability than patients receiving standard chemotherapy regimens (median overall survival 27 months vs. 17 months, *P* < 0.05). A limitation of our real-world approach is that we cannot rule out that the clinical benefit in the subgroup of patients receiving a molecularly matched treatment may be related to a selection bias for patients whose iCCA display a less aggressive clinical course. It could also be possible that particular genetic alterations, although not observed here, may have prognostic significance regardless of the targeted treatment. Even if this were the case our umbrella approach would be able to identify patients that have a higher probability to benefit from targeted or non-targeted treatment lines, respectively. While a complete and lasting treatment remission was only observed in one patient suffering from Lynch syndrome due to a germline mutation in the *MSH2* gene, who was treated with an immune checkpoint inhibitor, the overall response rate was 30% (*n* = 6/20) and the achieved disease control rate was 75% (*n* = 15/20) indicating that precision oncology may become a new therapeutic standard in the palliative care of iCCA patients. Noteworthy, a time-to-progression of more than 6 months was noted in 42% of patients (*n* = 8/19), in which mutations in the *BAP1*, *BRAF*, *BRCA1*, *ERBB2* and *PIK3CA* genes as well as fusions involving the *FGFR2, MET* and the *NRG1* gene were detected. Thus, 75% of iCCA patients in whom a targeted therapy was successfully translated benefited from our approach in terms of increased survival.

Fortunately, the targeted treatment of patients with iCCA is evolving rapidly. Several therapies that were experimental off-label treatments or administered within clinical studies in our cohort are now already standard treatment. Infigratinib (EU/3/20/2329) and pemigatinib (EMEA/H/C/005266) have been licensed for iCCA patients with *FGFR2* fusions. Entrectinib (EMEA/H/C/004936) and larotrectinib (EMEA/H/C/004919) are licensed for patients with solid tumours harbouring *NTRK* fusions that can also be present in iCCA patients [29], immune checkpoint inhibitors for tumours with high microsatellite instability (MSI-high) (EMEA/H/C/003820; EMEA/H/C/003985). A Phase II study has shown efficacy of combined dabrafenib plus trametinib treatment in patients with *BRAF*^*V600E*^-mutated biliary tract cancer [30]. Further drugs are in promising studies, e.g. zenocutuzumab (NCT02912949) and afatinib (NCT04410653) for patients with *NRG1* fusions. Thus, broad molecular profiling seems warranted for every iCCA patient with an indication for systemic therapy.

While both DNA and RNA sequencing results can be provided in a reasonable period of time in the vast majority of patients, the quality of the biopsy specimen (in particular the amount of tumour tissue included) may lead to failure of translocation detection in few patients. In one of our patients (#58), an oncogenic FGFR2 fusion was only recorded after sequencing of a re-biopsy. Considering that gene fusions occur in about 20% of iCCA patients, a lack of informative RNA sequencing data (e.g. as determined by AMP or TST170) may be considered as an indication for re-biopsy in patients eligible to an NGS-informed therapy.

A limitation of precision oncology is acquired therapy resistance. For instance, FGFR2 inhibition universally induces kinase domain mutations. In such a situation a re-biopsy of a progressive lesion may unravel the mechanism of resistance and allow to guide another line of targeted therapy (e.g. switch to another FGFR inhibitor or a combination therapy [31, 32]). Although re-biopsy is part of our diagnostic approach and is performed in patients still being amenable for further systemic tumour treatment upon progression, our own experience with cholangiocarcinoma is currently limited. As the LCCH umbrella is ongoing, we expect future relevant data regarding this important topic.

The translation of molecular findings into the treatment of iCCA patients faces some limitations. The number of approved drugs for precision oncology of cholangiocarcinoma is limited (even when drugs approved for other tumour entities are considered). In particular, *IDH1* mutations were detected in 16 patients, but ivosidenib could not be applied in our cohort as its EMA application was withdrawn and thus health insurances in Germany did not cover the costs for this treatment. However, the recently published data demonstrating the clinical benefit of ivosidenib for iCCA patients with *IDH1* mutation may have positively changed the situation [12].

Since November 2021, iCCA patients amenable to precision oncology at our institution can be referred to the Centre for Personalised Medicine Heidelberg (https://zpm-verbund.de/en/about-the-zpm/personalized-oncology/). Here, a specialised molecular tumour board provides standardised recommendations for NGS-informed therapy options and the associated network provides improved access to molecularly stratified studies.

The chance for successfully implementing an off-label therapy also depends on the time between molecular testing, recommendation of personalised therapy, and (in the case of off-label therapy) its approval by the health insurance. Here, a dedicated infrastructure like the Centre for Personalised Medicine Heidelberg may provide a framework for fast initiation of off-label personalised therapies, which may help to reduce dropout rates.

Furthermore, the information on the biological as well as the clinical impact of many detected variants remains incomplete. About 37% of variants (*n* = 58/156) detected in our study were categorised as variants of unknown significance (VUS). This does not necessarily indicate that patients, in whom VUS are detected, do not respond to a matched treatment, but, in light of the small number of approved drug options, reduces the likelihood of a positive decision on cost coverage by the patient´s health insurance. Another major bottleneck is the optimal timing of the molecular profiling and decision-making for targeted treatment. Due to approval of targeted treatment as second-line therapy so far, molecular testing is often initiated only after progression under first-line treatment, leading to deterioration of a significant number of patients until testing and therapy decision-making is completed. In 15% of patients, an NGS-informed therapy could not be implemented due to a deteriorated performance status. Of note, there was an overlap between the recruitment period of this study and the COVID-19 pandemic. As a consequence, regular clinical visits during outpatient care were replaced by remote visits (e.g. phone calls). Thus, the performance status of some patients may have been overestimated before deciding for molecular testing. It can be assumed that this factor will be eliminated in the post-pandemic area. However, there are clinical situations (e.g. progression of peritoneal carcinomatosis), in which rapid deterioration of the performance status naturally occurs. Ideally, broad molecular testing is initiated as soon as systemic treatment is considered. This may increase the fraction of patients that may benefit from targeted treatment. Of note, studies that explore targeted treatment as first-line therapy instead of chemotherapy are already ongoing, e.g. the PROOF study (NCT03773302) with infigratinib and the FIGHT-302 study with pemigatinib (NCT03656536) for iCCA patients with FGFR2 fusions.

In conclusion, iCCA represents a promising entity for precision oncological approaches, like our LCCH umbrella, and under real-world treatment conditions. Given the retrospective nature of evaluating treatment decisions in a single tertiary care centre, the promising data presented here need to be interpreted with caution. Our findings highlight the importance of registry studies to evaluate new treatment options in a real-world scenario and to compile sufficient data to address other clinically relevant questions (e.g. the possible predictive power of certain genetic alterations for therapy response, not only in the setting of molecularly matched but also conventional treatment regimens), which would be best performed prospectively across multiple centres.

## Supplementary information

Supplementary legends

Supplementary Figure 1

Supplementary Figure 2

Supplementary Figure 3

Supplementary Table 1

Supplementary Table 2

Supplementary Table 3

Supplementary Table 4

aj-checklist

STROBE_checklist

## Data Availability

Data are provided as Supplementary Material. Additional data are available upon reasonable request.
