# Supplementary material for: Precision oncology for intrahepatic cholangiocarcinoma in clinical practice
Source: Br J Cancer. 2022 Aug 19;127(9):1701–8. doi: 10.1038/s41416-022-01932-1 (PMC9390961; doi:10.1038/s41416-022-01932-1)
Supplement: Supplementary file 1 — Supplementary legends [file 41416_2022_1932_MOESM1_ESM.docx]

**Supplementary Figure and Table Legends**

**Supplementary Figure 1**: Panels used for DNA and RNA sequencing.

**Supplementary Figure 2**: Gene fusions detected by RNA sequencing (n=21).

**Supplementary Figure 3**: All genomic alterations detected among patients with intrahepatic cholangiocarcinoma (n=101). The given frequencies were calculated based on the number of patients with both available DNA and RNA sequencing data (n=74). The type of alteration is color-coded.

**Supplementary Table S1:** Characteristics of the study cohort at the time of inclusion

**Supplementary Table S2:** Sequencing platforms applied and genetic alterations detected in 101 iCCA patients

Abbreviations: ampl, high-level amplification; Archer, Archer Solid Tumor Panel; del, deletion; MASTER, NCT MASTER program; OCAv3, Oncomine Comprehensive Assay version 3; NA, not analysed; TSO500, Truesight Oncology 500; TST170. Truesight Tumor 170.

Class 3 variants (variant of unknown significance) are highlighted in yellow.

**Supplementary Table S3:** Targetable molecular alterations in iCCA patients.

Abbreviations: EMA, European Medicines Agency, ESCAT, ESMO scale for clinical actionability of molecular targets; ESMO, European Society of Molecular Oncology; FDA, U.S. Food and Drug Administration; MSI, microsatellite instability, TKI, tyrosine kinase inhibitor.

**References**

ESCAT variant classification: Mateo J, Chakravarty D, Dienstmann R, et al. A framework to rank genomic alterations as targets for cancer precision medicine: the ESMO scale for clinical actionability of molecular targets (ESCAT). Ann Oncol 2018;29:1895–902.

NCT variant classification: Horak P, Klink B, Heining C, et al. Precision oncology based on omics data: the NCT Heidelberg experience. Int J Cancer 2017;141:877–86.

**Supplementary Table S4:** Overview on targeted treatments and parameters on clinical outcome.
