# Supplementary figures and images for: Precision oncology for intrahepatic cholangiocarcinoma in clinical practice
Source: Br J Cancer. 2022 Aug 19;127(9):1701–8. doi: 10.1038/s41416-022-01932-1 (PMC9390961; doi:10.1038/s41416-022-01932-1)

DNA analyses

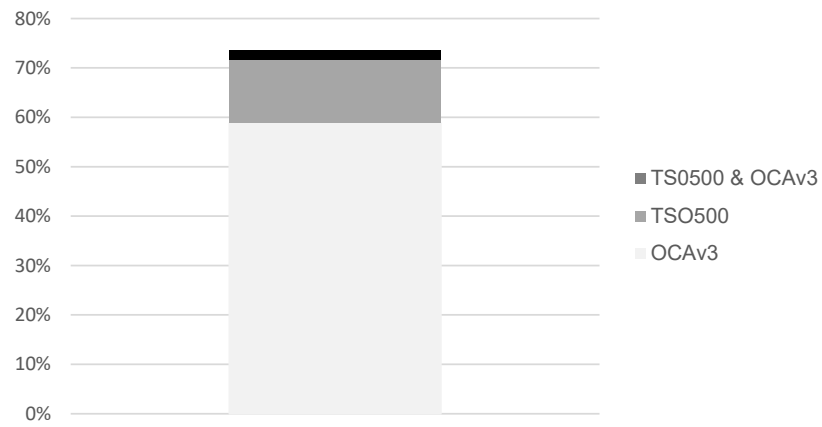

RNA analyses

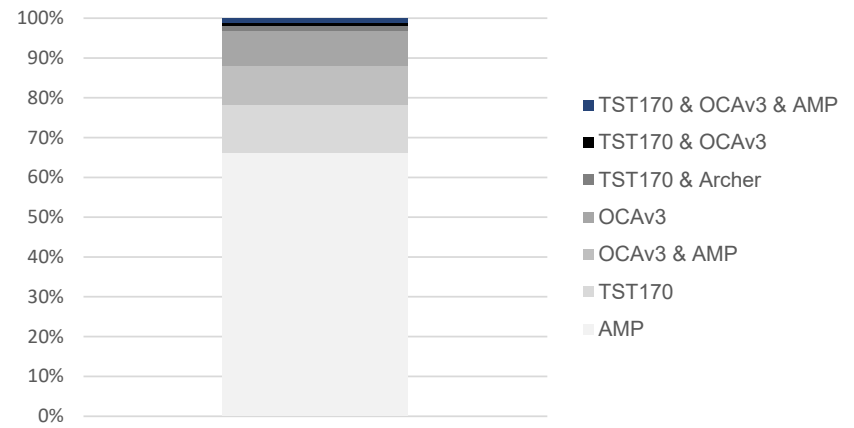

Supplement: Supplementary file 2 — Supplementary Figure 1 [file 41416_2022_1932_MOESM2_ESM.pdf]

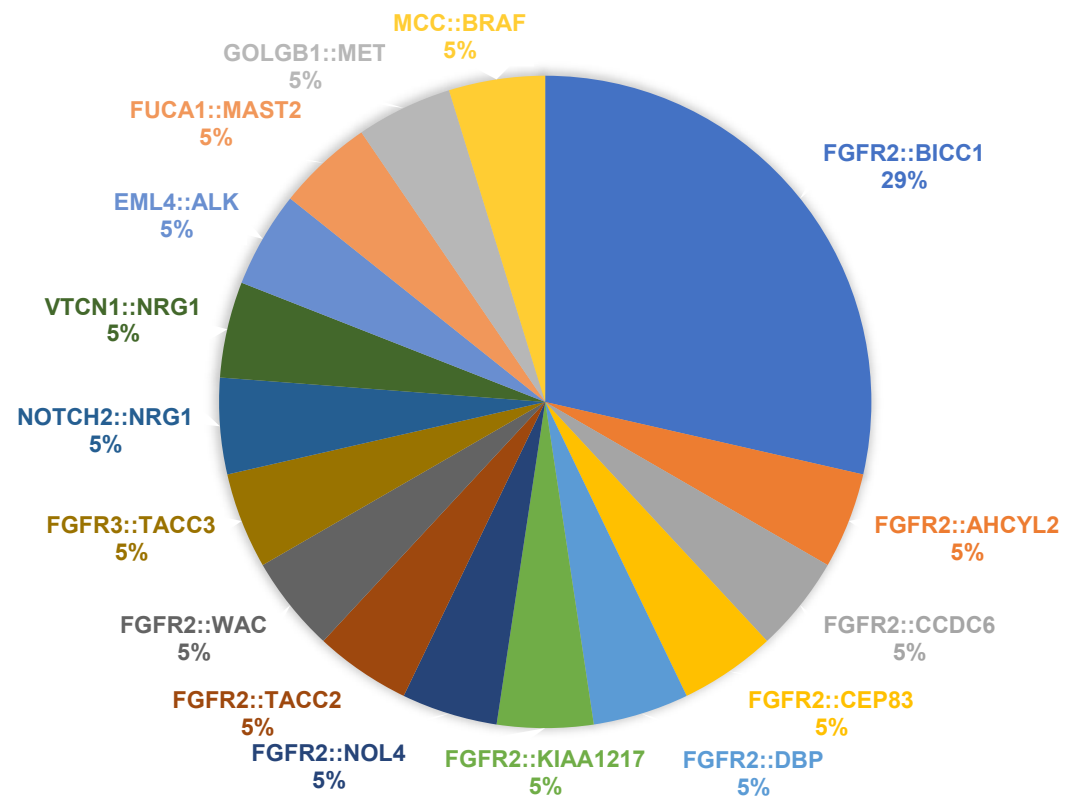

Supplement: Supplementary file 3 — Supplementary Figure 2 [file 41416_2022_1932_MOESM3_ESM.pdf]

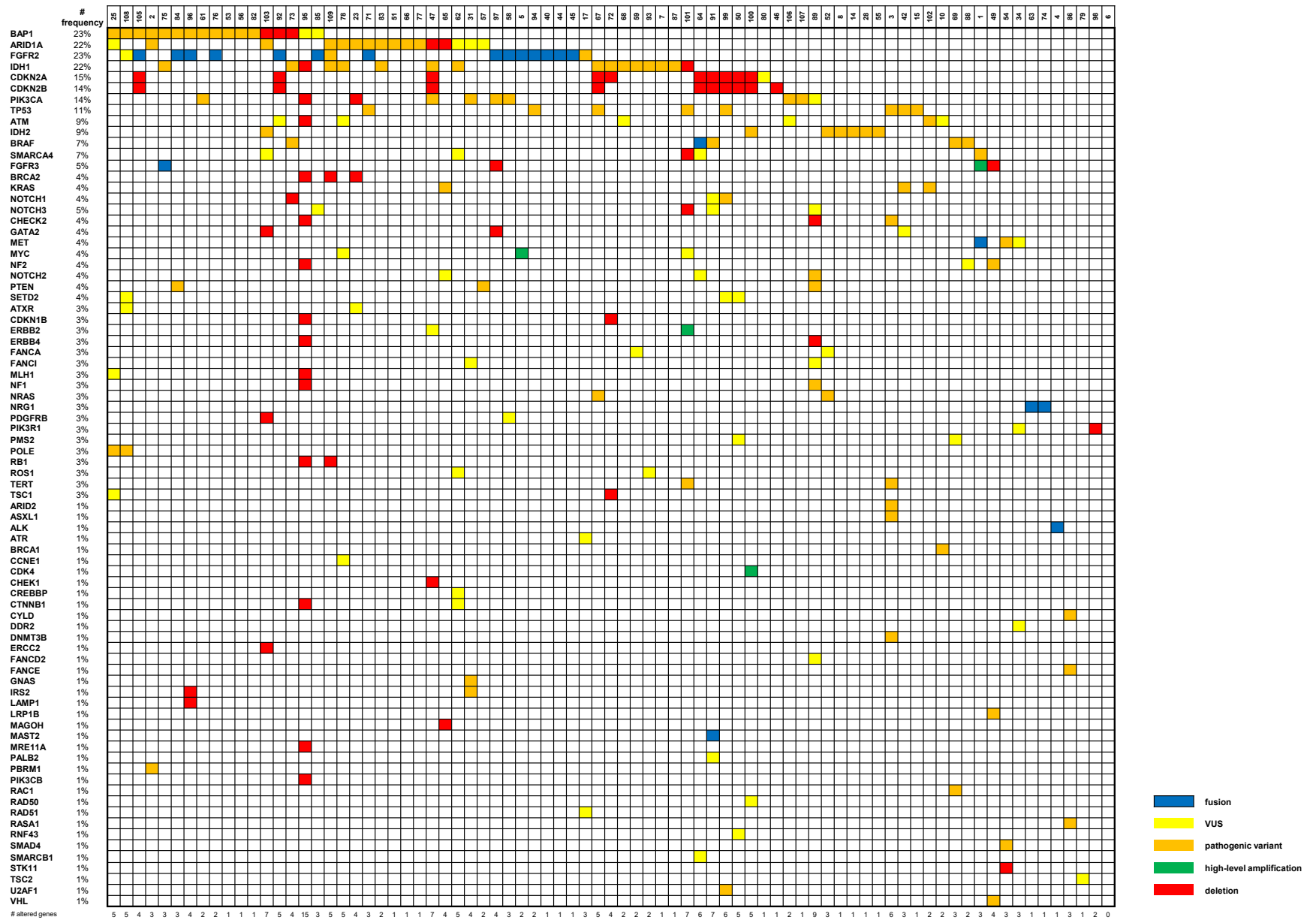

Supplement: Supplementary file 4 — Supplementary Figure 3 [file 41416_2022_1932_MOESM4_ESM.pdf]
